# Three-Dimensional Transesophageal Echocardiography in Percutaneous Catheter-Based Cardiac Interventions

**DOI:** 10.3390/jcm12175664

**Published:** 2023-08-31

**Authors:** Juan M. Farina, Timothy Barry, Reza Arsanjani, Chadi Ayoub, Tasneem Z. Naqvi

**Affiliations:** Department of Cardiovascular Medicine, Mayo Clinic, Phoenix, AZ 85054, USA

**Keywords:** three-dimensional echocardiography, transesophageal echocardiography, percutaneous, mitral valve repair, tricuspid valve repair, valve replacement, appendage closure device, paravalvular leak closure

## Abstract

Cardiac structural and valve interventions have remained surgical procedures for several decades. The ability to directly visualize the region of interest during surgery made imaging of these structures pre- and postsurgery a secondary tool to compliment surgical visualization. The last two decades, however, have seen rapid advances in catheter-based percutaneous structural heart interventions (SHIs). Due to the “blind” nature of these interventions, imaging plays a crucial role in the success of these procedures. Fluoroscopy is used universally in all percutaneous cardiac SHIs and helps primarily in the visualization of catheters and devices. However, success of these procedures requires visualization of intracardiac soft tissue structures. Due to its portable nature and rapid ability to show cardiac structures online, transesophageal echocardiography (TEE) has become an integral tool for guidance for all percutaneous SHI. Transcatheter aortic valve replacement—one of the earliest catheter-based procedures—while initially dependent on TEE, has largely been replaced by preprocedural cardiac CT for accurate assessment of valve sizing. Developments in echocardiography now allow live three-dimensional (3D) visualization of cardiac structures mimicking surgical anatomy during TEE. Besides showing actual 3D intracardiac structures, 3D-TEE allows visualization of the interaction of intracardiac catheters and devices with soft tissue cardiac structures, thereby becoming a “second pair of eyes” for the operator. Real-time 3D-TEE now plays an important role complementing multiplane two dimensional and biplane TEE during such interventions. In this review, we discuss the incremental role of 3D-TEE during various SHIs performed today.

## 1. Introduction

There has been enormous progress in catheter-based structural heart interventions (SHIs), with widespread use in the cardiology community. This has been made possible as a result of the development of new devices with improved performance as well as significant advances in the cardiovascular imaging field [1]. Fluoroscopy and two-dimensional (2D) transesophageal echocardiography (TEE) have been traditionally used for the guidance of SHI; however, these techniques are limited by their biplane projections of a complex three-dimensional (3D) cardiac structure [2]. In addition, the use of fluoroscopy is inadequate for the appropriate characterization of cardiac soft structures, while for 2D-TEE, multiple views and probe manipulations are usually necessary to display devices and the adjacent cardiovascular structures [3]. TEE technology has now advanced from biplane to 3D imaging, which allows for improved anatomic visualization and orientation of cardiovascular structures and their relationship with intracardiac catheters and devices [4].

This 3D approach may improve the interventional cardiologist’s ability to interact with the TEE images during SHI. Among many advantages, 3D-TEE images can reconstruct a more realistic anatomic display of cardiac chambers and valves in real time and provide critical intraprocedural information [5]. Therefore, 3D-TEE is expected to be a useful complementary imaging tool that supplies an interactive display for the operator to better understand the underlying pathology and plan the appropriate intervention, potentially improving procedural outcomes [6]. The 3D approach could better identify concomitant lesions, pitfalls, and procedural complications during SHI, including acute valve regurgitation, new wall motion abnormalities due to coronary occlusion, and perforation/dissection of cardiac structures. On a negative note, it has been reported that the growing use of 2D-TEE in SHIs could carry more TEE-related complications, which are probably associated with the need for several probe manipulations during these interventions [7,8]. With the use of 3D-TEE, catheters and devices can be detected and displayed even using a single 3D view, thus avoiding imprudent probe manipulations. 

The aim of this review is to analyze the current role of 3D-TEE imaging for SHI, focusing on the more common procedures that are FDA approved in the United States in which this rapidly evolving imaging technique can better assist the heart team in ensuring the best outcomes for patients. Non FDA approved devices currently undergoing phase three clinical trials including mitral and tricuspid valve replacement are also covered. 

## 2. Trans-Septal Puncture

Trans-septal puncture is a common procedure for SHI involving left-side cardiovascular structures. Some general rules need to be followed during this procedure; however, the majority of SHIs require a site-specific puncture for interventional success. Although trans-septal puncture is usually performed using only fluoroscopy, intraprocedural 3D-TEE could provide guidance for a more accurate targeted puncture and can be complementary to fluoroscopy and 2D-TEE imaging. Intracardiac ultrasound (ICE) may also be used to guide trans-septal puncture but requires the use of an additional catheter and offers limited 3D capability.

The 3D technique could also help to avoid iatrogenic trauma, to ensure alignment of devices and catheters, and to secure adequate space to maneuver within the left atrium [9]. Although the use of 3D-TEE is still limited in clinical practice during trans-septal puncture, it can also offer a more precise understanding of the trans-septal needle position in relation to surrounding cardiac structures, especially for under-skilled users in the presence of complex anatomy, and can potentially avoid complications including perforation of the right and/or left atrium or the aorta [1,9].

Recommended 3D-TEE imaging reconstructions to display the interatrial septum correspond to fluoroscopy projections: in the anteroposterior projection, the septum is displayed in an oblique perspective corresponding to the 3D-TEE oblique perspective from the right atrium; the right anterior oblique projection corresponds to the 3D-TEE en face view from the right atrium—by using this view, interventional cardiologists can follow the catheter tip from the superior vena cava to the fossa ovalis; in the left anterior oblique projection, the side-on profile of the septum corresponds to the 3D-TEE lateral perspective [1].

**Figure 1:**
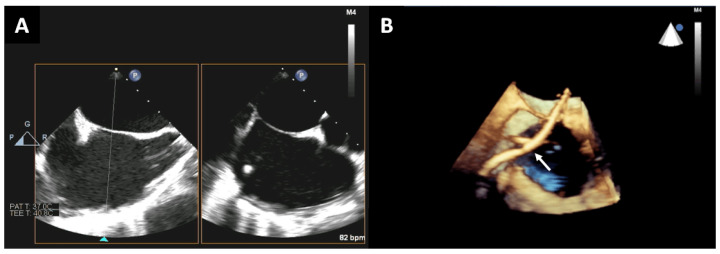
After obtaining the 2D-TEE bicaval view (**A**), multiplane reconstruction including the entire interatrial septum and surrounding structures should be performed (**B**). This reconstruction allows us to better appreciate the spatial relationship of the interatrial septum with the surrounding cardiac structures, and to better follow the catheter (white arrow).

When the catheter is facing the interatrial septum, the puncture site is traditionally recognized by the tenting [3]. This approximation of the catheter towards the interatrial septum can be guided by the 2D-TEE bicaval view, in which the septum is usually visualized and the tenting can be appreciated. Multiplanar reconstructions from this view can be particularly useful (Figure 1). In a complementary manner, the tenting can also be better observed by creating a 3D volume dataset in the lateral perspective.

## 3. Cardiac Valve Interventions

### 3.1. Transcatheter Aortic Valve Replacement

**Figure 2:**
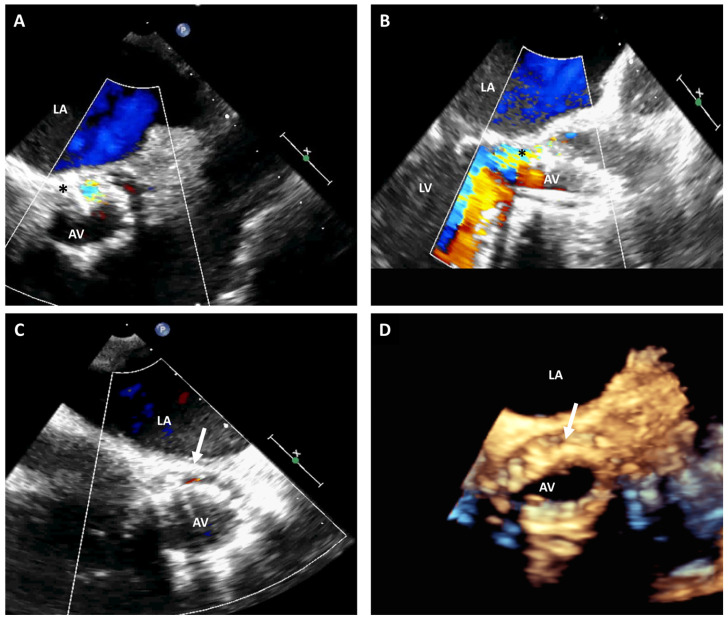
TEE short-axis and long-axis views of a CoreValve showing incomplete expansion of the posterior aspect of the valve causing paravalvular leak (asterisk) (**A**,**B**). A 2D-TEE short-axis image post balloon dilatation showing trivial paravalvular leak (white arrow) (**C**). Real-time 3D-TEE image at a lower plane than (**C**), showing flattening of the posterior aspect of the CoreValve due to nodular calcification causing underdeployment (white arrow) (**D**). LA: left atrium; LV: left ventricle; AV: aortic valve.

Recently, there has been an expanding increase in the availability and utilization of transcatheter aortic valve replacement (TAVR) [10]. The majority of these interventions are currently performed under conscious sedation without TEE guidance since preprocedural computed tomography (CT) scans provide detailed anatomic information on the aortic annulus, aortic valve, coronary arteries, and aortic root anatomy. Intraprocedural TEE can still provide unique benefits during procedural planning and implementation, particularly if the preprocedural CT scan is not conclusive or unavailable (Figure 2) [11]. This is closely related with the capability of 3D-TEE to accurately measure the aortic annulus size, define relevant adjacent anatomy, and promptly identify potential complications [12].

The aortic annulus is an ovoid shape that is less accurately sized by 2D-TEE imaging planes alone. A contemporary meta-analysis explored the precision of 3D-TEE in measuring aortic annular area, perimeter, diameter, and left ventricular outflow tract pre-TAVR [13]. The correlations between 3D-TEE and multidetector computed tomography (CT) measurements were strong with nonsignificant differences. Similarly, a recent publication evaluated the viability of decreasing renal injury by avoiding preintervention CT angiography and solely using 3D-TEE guidance in TAVR patients with established renal diseases [14]. In this study, the overall use of contrast was notably lower in the 3D-TEE group compared with the CT group with no significant differences in imaging and clinical endpoints. Therefore, 3D-TEE may be a promising strategy for pre-TAVR evaluation, especially in patients with kidney disease, in whom iodinated contrast exposure needs to be minimized.

During the procedure, precise positioning of the bioprosthetic aortic valve is critical. Although fluoroscopy plays the main role, the use of 3D-TEE can help with confirming the correct placement during valve deployment and minimizing the need for contrast injection. The differentiation between the valve and the underlying balloon relies on the detection of an echo-dense, sharp-edged structure [15]. In complex cases, 3D-TEE may improve the visualization of the stent [15]. The use of fusion techniques to overlay 3D-TEE and fluoroscopy can outline the annulus with higher precision [12,16]. It is critical to consider that proper positioning and deployment will depend on the type of device used (balloon-expandable, self-expanding, and mechanically expanded), and each case should be tailored according to the valve size, the characteristics and dimensions of the aortic root, and the location of the left main coronary artery [15,17].

After deployment, it is important to examine if complications have occurred, particularly prosthetic aortic regurgitation (AR). If 2D methods fail to accurately determine the severity of post-TAVR AR, color Doppler 3D volumes can help estimate the effective regurgitant orifice area (EROA) and the 3D color Doppler planimetry of the vena contracta area [18]. This real-time 3D information could facilitate the decision-making process with regard to the need for postdilatation and can provide clarity regarding the mechanism of AR (transvalvular or paravalvular) [19].

Following valve deployment, it is also important to explore mitral valve (MV) morphology and function. A low deployment in the left ventricular outflow tract (LVOT) can cause impingement of the anterior mitral annulus and the anterior leaflet deriving in pseudo-stenosis or mitral regurgitation (MR), which can also be assessed with 3D-TEE if conventional parameters are inconclusive. However, there may be an improvement in pre-existing secondary MR following TAVR, predominantly related to changes in left ventricular hemodynamics and mitral leaflet tethering [20]. A study using 3D-TEE techniques showed that MR improved in 54% of patients post-TAVR due to a reduction in tenting area and tenting height, and an increase in coaptation length [21].

Post-TAVR complications involving the aorta or left ventricle are rare but could be potentially catastrophic [22]. Real-time 2D complimented by 3D-TEE can rapidly diagnose complications such as contained or non-contained aortic annular rupture, compromise of coronary blood flow, and trauma to the mitral apparatus and is able to accurately characterize the extent of aortic dissection and involvement of neighboring structures [23].

### 3.2. Mitral Valve Interventions

The complexity of the MV apparatus and the proximity of the transcatheter devices with other cardiac structures could lead to difficulties during MV catheter-based interventions [24]. While conventional 2D echocardiography could have an inability to evaluate the complex anatomy of the MV apparatus, 3D-TEE has improved the understanding of the morphological and functional changes induced by MV percutaneous interventions, increasing their safety, reproducibility, and reliability [25].

#### 3.2.1. Edge-to-Edge Mitral Valve Repair

Real-time 3D-TEE imaging has fundamentally revolutionized the MV transcatheter edge-to-edge repair (TEER) procedure using MitraClip devices by facilitating the procedure, shortening the procedural time, and improving results. First, as already mentioned, successful trans-septal puncture requires a 3D understanding of the interatrial anatomy and its relationship to the surrounding structures. Obtaining an accurate view of the puncture site could be challenging using only 2D images, especially when the site is very posterior [26]. Thus, 3D-TEE may help identify the optimal area to be punctured, which can be slightly different in patients with different MR etiologies. In general, an appropriate distance (not inferior to 4.0 to 4.5 cm) between the septal tenting and MV orifice allows enough room for navigating the delivery system [3,26]. To measure this distance, a lateral perspective of the left side of the interatrial septum should be obtained [3,26].

The maneuvering of the delivery system into the left atrium and its advancement toward the MV could be monitored by using 3D-TEE images from an oblique perspective of the interatrial septum. This view displays the relation between catheters, the atrial wall, and the interatrial septum, thus allowing the operator to maneuver the catheters without injuring cardiac structures [3].

The correct orientation of the clips in a perpendicular manner to the coaptation line is critical for the intervention success and adequate leaflet grasp. This requires visualization of the MV on the short-axis plane. Two-dimensional TEE can only show this view in the transgastric plane and oftentimes may not be able to show this view in the correct anatomical plane or only show the view limited by gastric air. Real-time 3D imaging of the MV with the probe at the mid esophageal level provides a superb view of the entire MV from the atrial perspective, including anterior and posterior leaflet scallops, anterior and posterior commissures, and mitral annulus. This 3D-TEE view enables imaging of the clips’ arms and the MV coaptation line; therefore, it should be used to ensure that clips are perpendicular to the coaptation line [3]. After the grasping of the clips, the MV view from the left ventricular perspective can help evaluate the proper insertion of the clips and identify residual orifices. Real-time 3D visualization of the clip orientation pre- and postgrasp generally represents the greatest incremental utility for 3D imaging in this procedure. 

**Figure 3:**
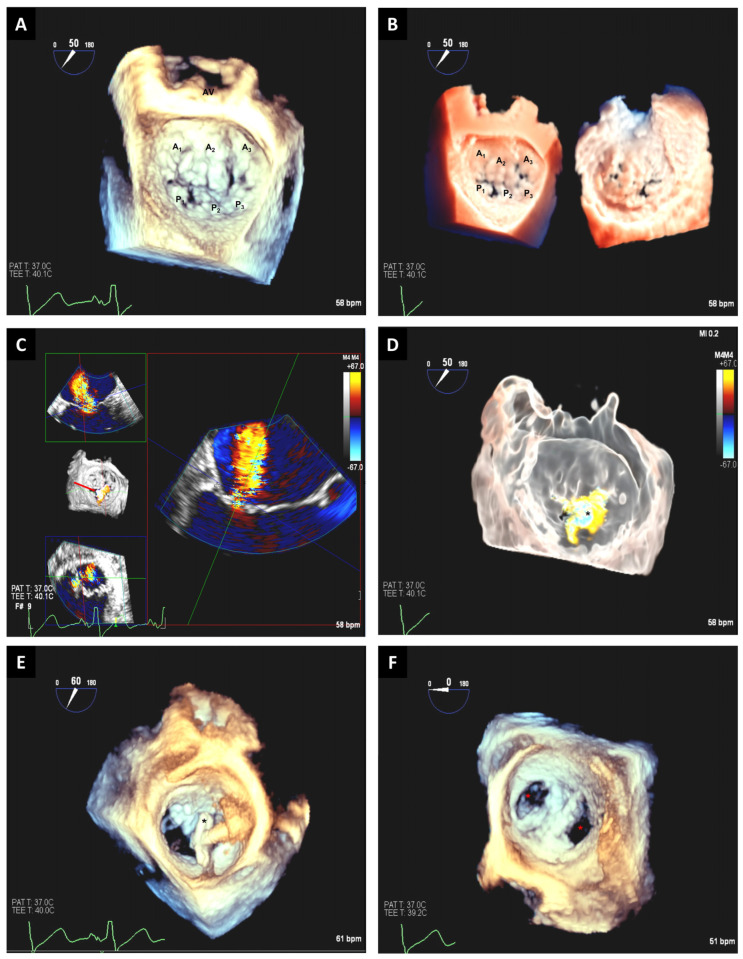
A 3D-TEE image of the mitral valve obtained in “zoom mode” showing mitral valve anatomy from the atrial perspective. Aortic valve is at 12 o’clock position. A large anterior leaflet with associated scallops (A1, A2, A3) and a short and restricted posterior leaflet with individual scallops (P1, P2, P3) are shown from lateral to medial position from the atrial perspective. Note coaptation gaps visible as black holes along leaflet coaptation and between P1 and P2 scallops (**A**). Same view of the mitral valve shown in 3D transillumination imaging (TI) in dual mode with atrial perspective on the left and ventricular perspective of the mitral valve on the right. This mode allows further image enhancement by allowing focused visualization of area of interest with “light” mode. Note the tenting of the mitral valve both from atrial and ventricular perspectives in TI imaging mode (**B**). Preprocedural 3D cine full volume loop in the color Doppler mode with multiple image reconstructions demonstrating severe secondary mitral valve regurgitation (red arrow) with restriction of the posterior mitral leaflet (**C**). The broad jet of mitral regurgitation can be seen across the coaptation margin (asterisk) in the 3D “glass” mode, which highlights further mitral valve tenting and the color Doppler mitral regurgitation jet origin (**D**). During deployment of MitraClip, 3D-TEE images are crucial to ensure proper orientation of the clips (asterisk) perpendicular to the coaptation line (**E**). Due to broad mitral regurgitation jet origin, two clips were needed, with the second placed lateral to the first clip. Postprocedural 3D-TTE images show double orifice (asterisks) mitral valve with two parallel well-deployed clips (**F**). AV: aortic valve.

**Figure 4:**
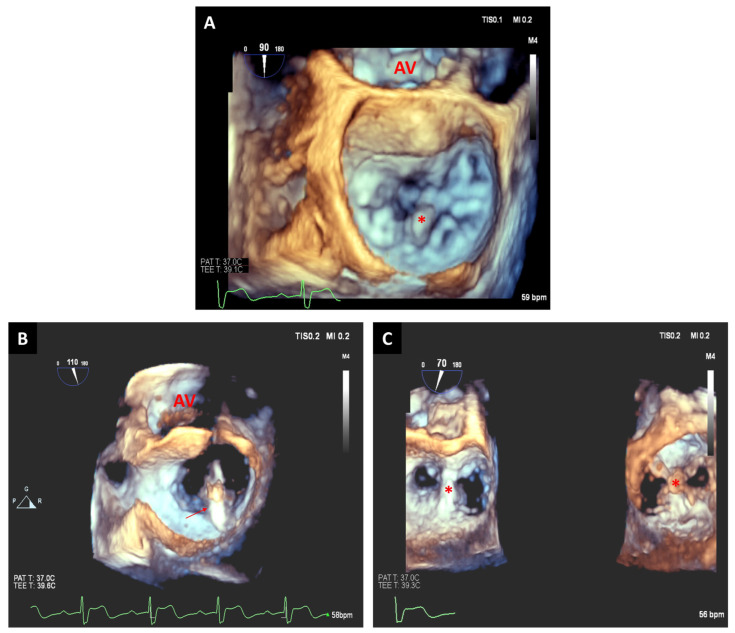
Real-time 3D-TEE image from the atrial perspective (Surgeon’s view) showing degenerative mitral valve with flail P2 scallop (asterisk) (**A**). A 3D-TEE image also from the atrial perspective during MitraClip placement showing the device arms (red arrow) situated at the A2/P2 interface with the mitral leaflets in the open position in diastole (**B**). A 3D-TEE image from the atrial (**left**) and ventricular (**right**) perspectives showing the mitral valve post MitraClip (asterisk) deployment at the A2/P2 interface and double orifice (**C**). AV: aortic valve.

Only a limited number of 3D-TEE views are normally required for the intraprocedural guidance; meanwhile, for 2D-TEE, various views are usually needed to establish the adequate position of the clip(s). Additionally, 2D-TEE can be inaccurate in locating the tip of the catheters and its spatial relationship with the coaptation line while, with 3D-TEE, the spatial location of catheters in relation to surrounding structures is easier to comprehend [27]. Therefore, the use of 3D-TEE could decrease time requirements for critical TEER steps and improve safety during these procedures (Figure 3 and Figure 4).

In a series of 26 patients, Altiok et al. demonstrated that 3D-TEE provided critical additional information compared with 2D-TEE in various central steps of the TEER intervention. Those steps included the guidance and maneuvering of the catheters, the accurate positioning and alignment of the first clip, the confirmation of correct grasping location, and the proper positioning of any additional clips, if required [28]. Real-time 3D full volume datasets allow rapid assessment of the direct planimeter area of the residual MV orifices without geometrical assumptions inherent in spectral Doppler. Color Doppler 3D-TEE images could also help visualize the residual MR jets, and the 3D vena contracta area may also allow the quantification of residual MR [29].

#### 3.2.2. Transcatheter Mitral Valve Replacement

**Figure 5:**
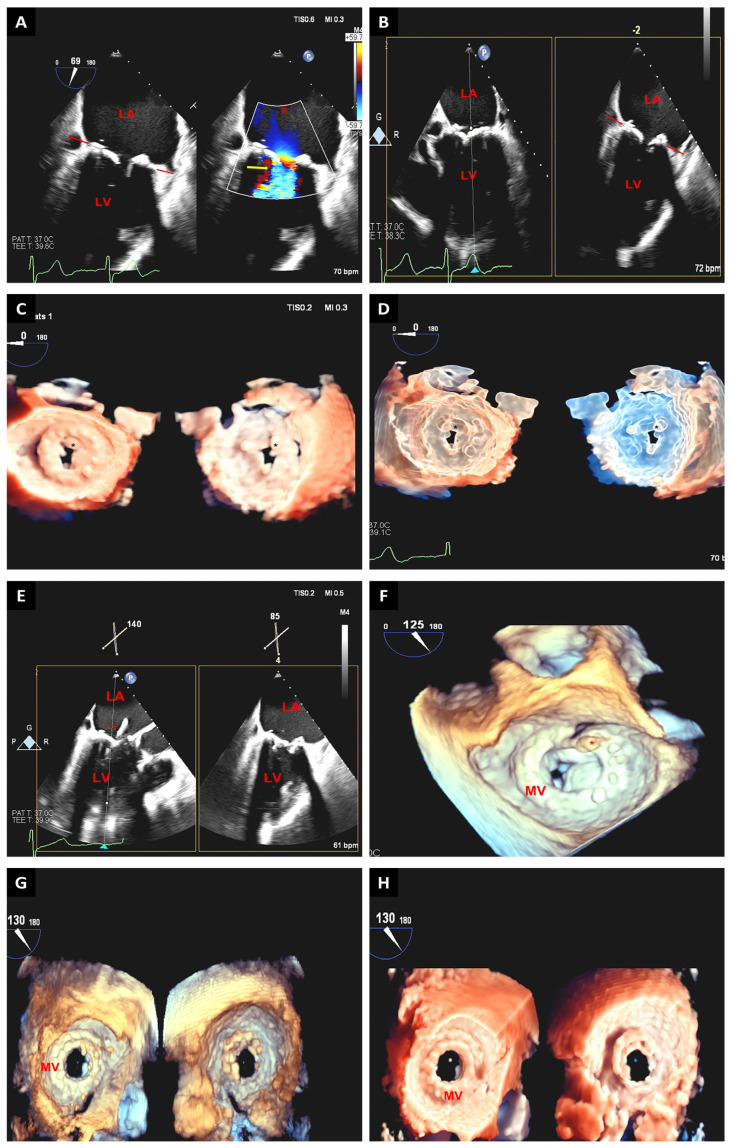
Presents 2D-TEE images of a bi-commissural view with and without color show severely thickened restricted mitral valve prosthetic leaflets (red arrows) with turbulent flow (yellow arrow) in diastole (**A**). The 2D-TEE biplane image shows severely restricted mitral valve prosthetic leaflets in diastole (red arrows); overall findings consistent with bioprosthetic stenosis secondary to degeneration (**B**). Real-time 3D-TEE images with a TrueView (**C**) and glass view (**D**) projection from the atrial (**left**) and ventricular (**right**) aspect show severely restricted prosthetic mitral valve leaflets (asterisk), confirming degenerative bioprosthetic stenosis. Intraprocedural 2D-TEE images demonstrating the guidewire across the mitral prosthesis (red arrow) prior to delivery of the new mitral prosthesis (**E**); 3D-TEE view from the atrial perspective (en face view) confirms the guidewire position (asterisk) between the anterior and lateral leaflets of the mitral prosthesis (**F**). Standard (**G**) and TrueView (**H**) 3D-TEE projections from the atrial (**left**) and ventricular (**right**) perspectives depict the status post 29 mm Edwards Sapien (valve-in-valve replacement). LA: left atrium; LV: left ventricle; MV: mitral valve.

Percutaneous annuloplasty or leaflet repair techniques may not be sufficient to eliminate MR in some scenarios depending on native valve characteristics [30]. Transcatheter mitral valve replacement (TMVR) has the potential to treat several mitral conditions, including mitral stenosis or MR cases not suitable for TEER procedure. The TMVR procedure has also been used to perform valve-in-valve reinterventions to replace prior degenerated prostheses (Figure 5) [31].

However, TMVR procedures are highly challenging due to several factors. These include the complex geometry of the mitral apparatus, the large size of the mitral annulus, the relative lack of calcification (which is a landmark used for fluoroscopic guidance), and the need to protect neighboring structures such as the coronary sinus, coronary arteries, and the LVOT [30]. In order to achieve a successful intervention, a comprehensive examen of the mitral apparatus, the level of calcification, and the relationship to adjacent structures is critical [32]. 

For preprocedural planning, 3D-TEE datasets centered on the MV can be reconstructed using different planes to perform thorough quantifications of the mitral annulus. One study confirmed the clinical feasibility and accuracy of the assessment of the MV geometry with 3D-TEE when compared to CT measurements [33]. In addition, the reconstruction of the en face views can provide a more realistic visualization of the anatomy and relationship of the MV components. 

Regarding the use of intraprocedural TEE, 2D- and 3D-TEE guidance could be important for successful valve deployment. The mid-esophageal short-axis and bicaval views allow us to display the interatrial septum and maneuver the catheters; the mid-esophageal commissural view can help achieve correct medial–lateral positioning of the device; the mid-esophageal long-axis view is useful to estimate the anterior–posterior positioning of the device; lastly, a transgastric short-axis view can evaluate coaptation of leaflets [30].

Postdeployment, 3D-TEE can be useful to verify the proper position and function of the device, to identify and quantify the severity of residual MR, and to detect procedural complications.

### 3.3. Paravalvular Leak Closure

Perivalvular dehiscence is an undesired complication that may happen after a prosthetic valve replacement procedure, more frequently affecting patients with mechanical mitral replacements. Transcatheter leak closure has emerged as a viable alternative to surgical redo procedures, especially in high-risk patients. However, these percutaneous procedures are usually complex and detailed imaging guidance is required [32,33].

**Figure 6:**
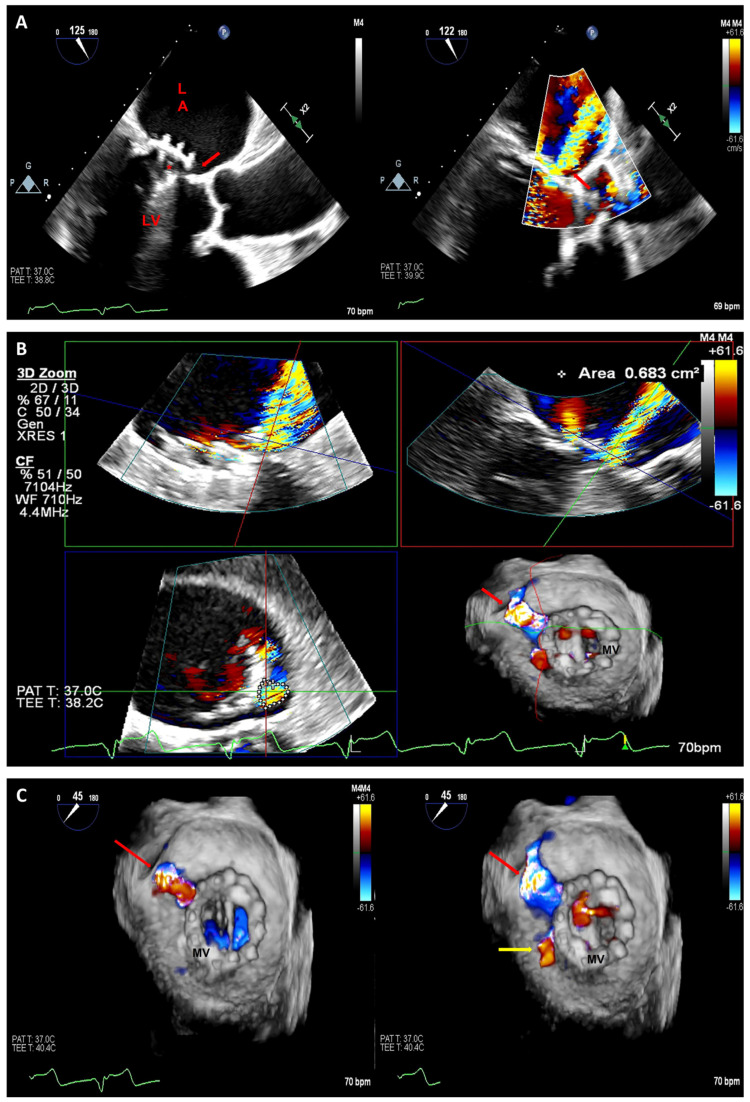
Two-dimensional TEE long-axis view with and without color Doppler demonstrating mechanical mitral prosthesis (asterisk) with anterolateral paravalvular leak (red arrow) (**A**). Three-dimensional multiplanar reconstruction confirming the anterolateral periprosthetic regurgitation (red arrow) (**B**). Real-time 3D-TEE color Doppler (atrial perspective) demonstrating anterolateral directed periprosthetic regurgitation adjacent to the mitral prosthesis at eleven o’clock (red arrow) (**C**). A smaller posterolateral defect (seen at nine o’clock) is also noted (yellow arrow). LA: left atrium; LV: left ventricle; MV: mitral valve.

**Figure 7:**
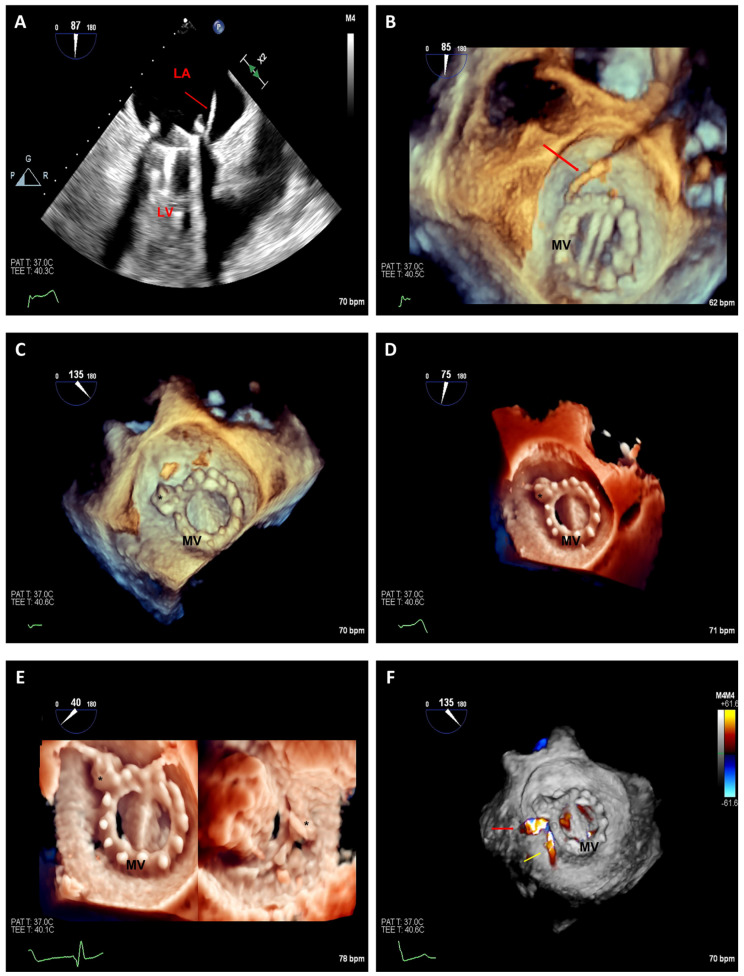
Two-dimensional (**A**) and 3D-TEE (**B**) images demonstrating a wire (red arrow) across the anterolateral paravalvular defect. Real-time 3D-TEE standard (**C**) and TrueView (**D**) images showing the closure of the previously illustrated anterolateral paravalvular leak with a 10 mm Amplatzer closure device (asterisk). TrueView images of the atrial (**left**) and ventricular (**right**) perspectives of the mitral prosthesis (**E**) confirming the closure of the periprosthetic regurgitation (asterisk); the smaller posterolateral defect was not closed. The 3D-TEE image from atrial perspective demonstrates two residual jets of periprosthetic regurgitation. One residual leak is slightly posterior to the placed closure device (red arrow) and the second jet is the posterolateral one previously identified at nine o’clock (yellow arrow) (**F**). LA: left atrium; LV: left ventricle; MV: mitral valve.

The 3D multiplane reconstruction can be particularly useful to confirm the location and extent of periprosthetic regurgitation. Intraprocedural 3D-TEE guidance may provide critical information to the interventional cardiologist when crossing the dehiscence, as imaging can confirm the guidewire position across the perivalvular defect. In particular, the use of multiplane 3D images can be important in cases with severe calcification, sinuous dehiscences, or complex locations [32]. The en face views of the valve could also provide critical advice for device sizing and achieving accurate placement (Figure 6 and Figure 7) [34].

Traditional 2D-TEE has several limitations in the assessment of dehiscences, including the interference of prosthetic material, sutures, or calcifications. However, the most challenging one is its inability to delineate the complete extent of periprosthetic dehiscences, which can be multiple and frequently anatomically complex [27]. In this sense, even when 3D-TEE shares some of these limitations with 2D techniques, a 3D approach could better define the exact morphological characteristics of the defect. 

Fusion between TEE and fluoroscopy imaging may simplify the closure procedure, particularly in cases of radiolucent prosthetic valves [35]. By complementing TEE, Doppler, and 3D modalities on fluoroscopy, fusion imaging may help to perform a site-specific trans-septal puncture, correctly identify the site of the leak, deploy the device appropriately, and detect potential interference with prosthesis [1].

One study investigated the clinical value of 3D-TEE for the catheter-based closure of paravalvular leaks in mitral prosthesis. This study showed no significant disagreement between 2D- and 3D-TEE color flow Doppler for the location of each leak, the vena contracta width, and the reduction in the paravalvular leak vena contracta width. However, only 3D-TEE had the ability to detect the decrease in the effective orifice circumferential length of the leak, suggesting that this technology could provide unique and additive information in this scenario [36].

A study of ten patients with persistent mitral leak following transcatheter closure demonstrated that 3D-TEE was able to confirm the location and number of the residual leaks, along with their morphological characteristics (shape, extent, and area). Additionally, 3D-TEE imaging also helped identify the exact location of the devices, allowing improved comprehension of the causes underlying the intervention’s failure [32].

In a study of 166 patients with either mitral or aortic paravalvular leaks, TTE, TEE, and 3D printing were utilized for the preoperative evaluation of the defects. Among the included cases, 68 used preoperative TEE and 3D printing and showed advantages when compared with traditional TEE guidance, with a decrease in interventional and fluoroscopic times [37].

### 3.4. Tricuspid Valve Interventions

Catheter-based treatments of tricuspid regurgitation (TR) are becoming a viable option for patients who are at high risk of surgical procedures. Therefore, different devices with diverse mechanisms of action have been developed [38]. Specific challenges related to transcatheter approaches include the dilatation and complex morphology of the tricuspid annulus, non-uniform annular tissue, the dimensions of right ventricle chamber, the thin right ventricle wall, and the proximity of the right coronary artery to the tricuspid annulus [39]. Therefore, a detailed understanding of the anatomy and pathophysiology of TR is critical for the selection of the most appropriate device for each patient [40]. The grading of TR severity could be enhanced by the use of 3D measurements of the effective TR orifice areas [41]. The 3D images can also help investigate if the pacemaker leads have a role in causing TR and if their spatial position could create potential complications during an intervention [42,43]. 

**Figure 8:**
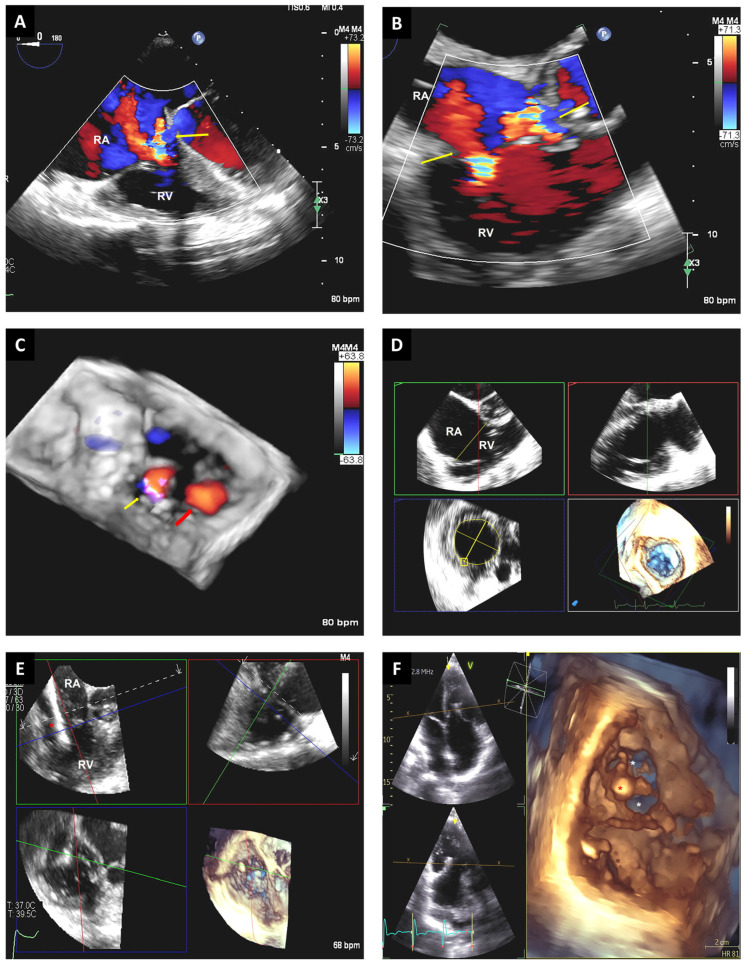
Preoperative 2D-TEE images at gastroesophageal junction of the tricuspid valve at 0° (**A**) and 70° (**B**) with color Doppler demonstrating tricuspid annular dilatation and severe tricuspid regurgitation (two jets, yellow arrows). Real-time 3D-TEE image with color flow confirming two jets of tricuspid regurgitation (**C**): one jet is located between posterior and septal leaflets (red arrow) and the second jet is located between the anterior and septal leaflets (yellow arrow). A 3D-TEE multiplanar reconstruction prior to tricuspid clip placement reveals annular dimensions (yellow line) and valvular area (yellow circle) (**D**). Intraprocedural placement of the delivery system (asterisk) entering the right ventricle by multiplanar reconstruction to identify the location of the delivery catheter between the anterior and septal leaflets (**E**). Postprocedural 3D-TEE images from the right ventricle perspective show double orifice (white asterisks) tricuspid valve with excellent clip deployment position (red asterisk) (**F**). RA: right atrium; RV: right ventricle.

The 3D multiplane imaging could be crucial during these complex cases. The 3D multiplane mode is usually most useful from the mid-esophageal commissural views at the site of the TR jet to display the four-chamber view, as well as from the transgastric short-axis tricuspid valve (TV) view. These views display the leaflet grasping view, which is critical to evaluate feasibility and to decide the final transcatheter device placement. These views also provide information to determine the TR mechanism, orifice size, septal leaflet length, and septal leaflet curling [44]. In addition, 3D-TEE multiplane reconstructions can also improve the understanding of TV anatomy, as en face visualization of the TV allows direct identification of the leaflet. In this way, the need to depend on the identification of adjacent structures could be overcome [44]. Oftentimes, multiple 3D datasets from various probe positions are required for a complete assessment of the TV and annulus. Considering the location of the TV, mid-esophageal, distal esophageal, shallow transgastric, and deep transgastric may be the most useful views for 3D multiplane reconstructions. The intraprocedural use of 3D technologies could reduce the need for additional images as the positioning of the transcatheter devices can be decided from the 3D en face views or multiplane reconstructions (Figure 8).

Lastly, 3D-TEE multiplane reconstructions can help with the final evaluation of the position of the device following placement and 3D color Doppler should assess the presence and magnitude of potential residual jets. For some particular devices, transgastric views with 3D reconstruction of the right ventricle anchor path and landing zone may be important [39].

### 3.5. Left Atrial Appendage Closure

With the FDA approval of left atrial appendage (LAA) catheter-based occlusion devices, the use of cardiovascular imaging for real-time assessment of the LAA orifice area has become critical for accurate sizing and placement of devices [45]. Previous evidence supports the use of 3D-TEE to ensure correct visualization and measurement of the LAA orifice [46]. A study of 137 patients demonstrated that 3D-TEE is more precise than 2D-TEE for the evaluation of LAA orifice size, with a strong correlation between 3D-TEE and CT measurements [47]. Another study of 22 patients undergoing LAA closure showed that 3D-TEE guidance performed during the interatrial septal puncture, the exchange of the sheath, and the release of the closure device was a reliable and effective imaging modality during this intervention [48]. In this last study, authors concluded that a full 3D-TEE view of the interatrial septum facilitated the determination of the optimal puncture site and avoided deformations of devices or significant residual leaks. Additionally, 3D-TEE helped define the proper position of the closure device with respect to the MV and the left superior pulmonary vein. Lastly, a study of 28 patients demonstrated that the evaluation of LAA anatomy by 3D-TEE helped select the most appropriate closure device and device operation during the procedure [49]. In this study, patients underwent both a preoperative 3D-TEE to comprehensively evaluate the LAA (anatomic features, landing zone dimension, and depth) and an intraprocedural 3D-TEE to guide the septal puncture, device maneuvering, and to assess results of the occlusion. No significant residual shunts, pericardial effusion, or displacement of the device were observed following the procedure. No closure-related complications were seen during follow-up [49].

**Figure 9:**
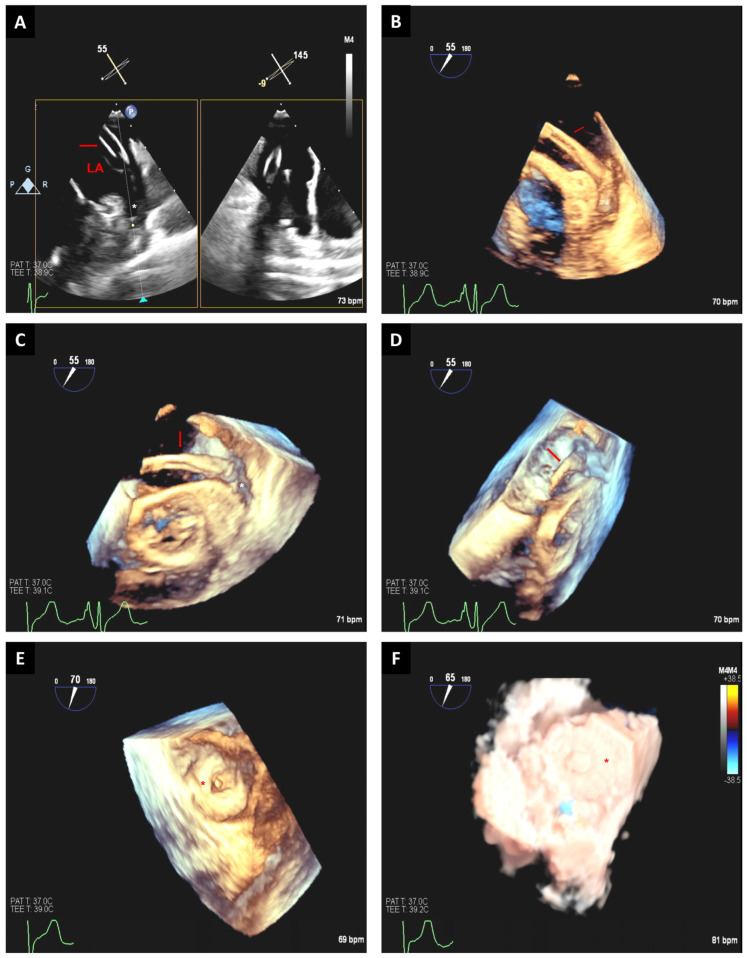
Mid-esophageal TEE images show 2D x-plane view at 55° and 145° (**A**) guiding the catheter (red arrow) into the left atrial appendage (asterisk). Real-time 3D-TEE multiplane reconstruction from the prior views can help guide the catheter (red arrow) into left atrial appendage (asterisk) (**B**–**D**). Postprocedure, using 3D-TEE images, the occlusion device is visualized and is well-expanded within the left atrial appendage (asterisk) (**E**), and no significant leak between the left atrium and left atrial appendage is noted when using TrueView techniques (**F**). LA: left atrium.

During transcatheter occlusion procedures, the 3D-TEE technique is usually used to ensure the proper position of the catheter by using an en face view of the LAA orifice [3]. A small angulation from the prior position can display the correct position of the catheter into the LAA, and this same view can be used for the expansion of the device. The visualization of the tip of the catheter and its spatial relation with the LAA can be observed from the long-axis view of the LAA (Figure 9) [3].

### 3.6. Pulmonary Veins Interventions

Pulmonary vein stenosis is considered an uncommon disease with a challenging diagnosis and potential undesired consequences if left untreated. Currently, injury from radiofrequency ablation for atrial fibrillation has become the main cause of this condition, and transcatheter intervention is the most common therapeutic approach. When this diagnosis is suspected, multimodality imaging methods are usually required for confirmation [50].

**Figure 10:**
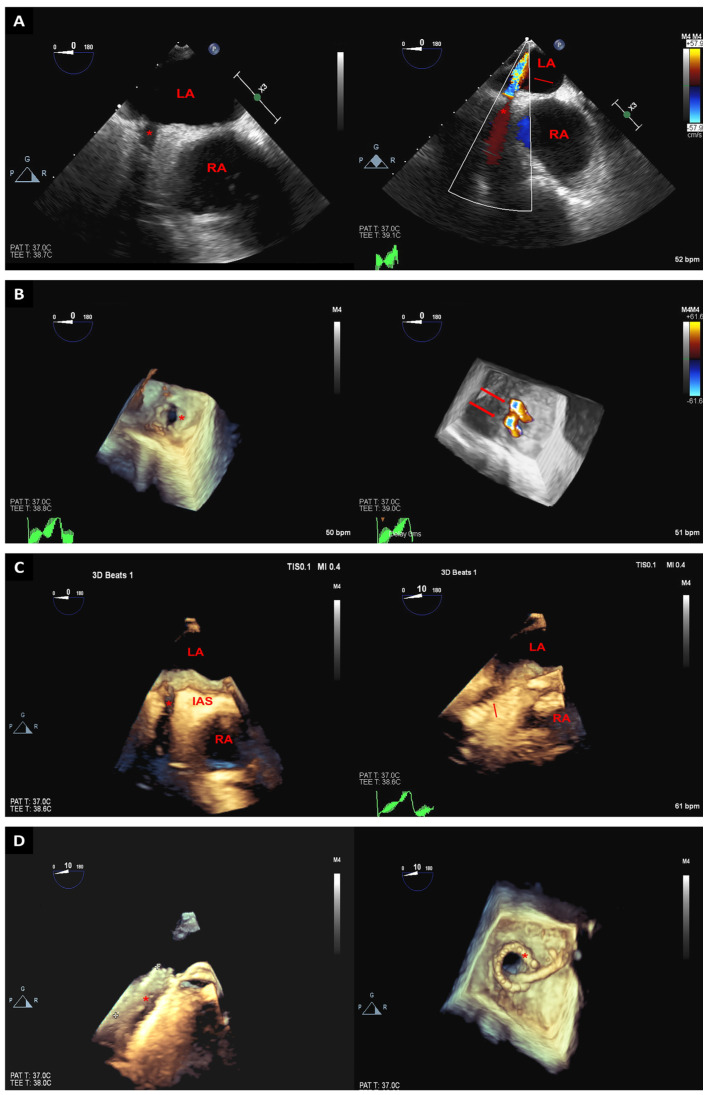
Two-dimensional TEE images with and without color Doppler of the right upper pulmonary vein (red asterisk). In the color Doppler images, the turbulent flow at the entrance of the right upper pulmonary vein into the left atrium (red arrow) suggests pulmonary vein stenosis (**A**). Real-time 3D-TEE images reveal a small central ridge at the orifice of the right upper pulmonary vein (red asterisk). Three-dimensional color-flow imaging demonstrates two jets of turbulent flow (red arrows) from the right upper pulmonary vein into the left atrium (**B**). Live 3D-TEE images of the right upper pulmonary vein (red asterisk), left and right atria, and interatrial septum. The catheter used in the procedure can be seen (red arrow) within the pulmonary vein (**C**). Status of 3D-TEE images post stent implantation in the right upper pulmonary vein from the long- and short-axis views (asterisk) (**D**). LA: left atrium; RA: right atrium; IAS: interatrial septum.

Angiography is traditionally utilized to locate pulmonary veins stenosis. Using only this technique, it could be difficult to easily identify occluded pulmonary veins; however, 3D-TEE could help locate them. The use of 3D-TEE during pulmonary vein interventions provides a real-time assessment of pulmonary veins anatomy along with a better understanding of the spatial relationships between cardiac structures [51]. These advantages provided by 3D technologies could help determine wire location and appropriate stent deployment (Figure 10) [51].

Another scenario for the potential use of 3D-TEE is to complement other imaging methods during anatomy-driven catheter-based radiofrequency pulmonary veins ablation [52]. A 3D-TEE view of the roof of the left atrium could allow the visualization of all the four pulmonary veins ostia in one image. Nonetheless, the right and left veins could be widely separated in some cases, and it could become complicated to display both the roof of the atrium along with the pulmonary veins. Thus, the evaluation of left and right pulmonary veins should be performed separately in those cases. 

The use of intraprocedural 3D-TEE has been also proposed in patients with Scimitar syndrome, characterized by an anomalous venous return from the right lung directly into the inferior vena cava. Full-volume 3D-TEE imaging can illustrate the abnormal pulmonary vein entrance into the inferior vena cava and can help identify the orientation of the scimitar vein with respect to the hepatic veins and the inferior vena cava [53]. This can be critical for interventional planning. Color flow Doppler 3D-TEE images can also identify a high-velocity jet from the abnormal pulmonary vein, which will be different from the laminar flow in the hepatic veins and inferior vena cava. 

## 4. Discussion

Fluoroscopy has been the traditional method for intraprocedural guidance in SHI and has its own strengths. Wires, devices, and catheters for SHI have been designed and developed to be radiopaque to maximize fluoroscopic guidance, while these medical tools usually create undesired artifacts in echocardiography imaging. Moreover, in contrast to echocardiography, fluoroscopy displays a larger field of view that allows interventional cardiologists to follow and visualize long segments of catheters. The temporal resolution of fluoroscopy is also appropriate to maneuver the catheters and devices, while a major limitation of 3D imaging is its poor temporal resolution, which is related to the increased use of data points to generate 3D images [40]. Therefore, an optimal interplay between temporal and spatial resolutions is critical when using 3D-TEE reconstructions for SHI.

As outlined in this review, multiplane 3D-TEE imaging can offer an interesting number of advantages when utilized in a complementary manner to fluoroscopy during SHI. Soft tissues of critical cardiac structures are transparent to radiography; so, echocardiography is of paramount importance for an accurate anatomical assessment of the targeted structures. When comparing the more novel 3D approach with traditional 2D techniques, real-time multiplanar 3D-TEE imaging, and particularly en face views, supply additive and more appropriate guidance of catheter manipulation by providing improved imaging and comprehensive information about the spatial positioning of wires and devices and their relationships with surrounding cardiac structures. The multiplanar perspective of 3D-TEE allows the use of several imaging planes specific to each procedure with no need for several changes in probe position. This may lead not only to more accurate technical outcomes but can also help in reducing complications and suboptimal deployments during SHI. Additionally, 3D-TEE techniques can help shorten the duration of procedures, thus reducing the volume of contrast and radiation exposure both for the patients and the heart team.

New technologies are continually being developed in the field of cardiovascular imaging. ICE has been proposed as an alternative for TEE guidance for SHI. However, this method is not without disadvantages, including costly single-use catheters, the potential interference between the catheter and temporary/permanent pacemaker leads, limited 3D evaluation (3D-ICE is restricted to a small volume compared with 3D-TEE), and smaller field of view, making it challenging to appropriately display structures far away from the right atrium [54]. Additionally, conventional 3D-TEE could not provide images with appropriate definition and depth perception in some scenarios. Considering that displaying 3D structures with 3D reconstructions on a 2D screen could be a challenging task, new tools are currently available to improve the perception of depth and the depiction of cardiovascular structures [55]. Transillumination (TI) is a novel technology that projects a virtual light source into the 3D images. The light source can freely move within the dataset to improve image features and depth and to highlight the structures of interest [55]. One study enrolled 30 patients with suboptimal conventional 3D images and demonstrated that TI improved the visualization of different cardiac structures and anatomic abnormalities [56]. The transparency or “glass” effect is another novel technique with several advantages such as the capacity to adjust tissue transparency, thus enhancing the representation of structures of interest; this tool demonstrated promising results compared with TI and conventional 3D technologies in the assessment of cardiac anatomy, border delineation, and pathogenetic mechanisms [57]. An additional benefit of this transparency technique consists in its capacity to combine 3D structural data with 3D color Doppler to better visualize the origin, location, and direction of pathological jets [55]. In one study including 30 patients with different cardiac diseases, the addition of the transparency mode to TI improved the diagnostic capabilities and clinical utility of 3D-TEE, thus supporting its potential to enhance TEE guidance during SHI [55]. 

As the use of 3D-TEE is becoming more frequent for cardiac SHI, the applications of this technique are now expanding to other percutaneous interventions. It has been proposed that 3D-TEE techniques could also have a role in planning or guiding percutaneous aortic procedures [58], as the use of 3D-TEE during cases of aortic dissection could enhance the anatomical delineation of the dissection. In particular, 3D imaging could better locate the entry site, discern true from false lumens, recognize potential coronary involvement, and identify the relation between the dissection flap and neighboring structures.

However, potential barriers exist for the general implementation of 3D-TEE imaging in the catheterization laboratory. First, interventional cardiologists are more familiar with fluoroscopy guidance than with echocardiography imaging for SHI. As 3D-TEE is becoming a useful complementary tool during SHI, training programs for interventional cardiologists could better include the use of this novel technique. Moreover, images obtained from fluoroscopy and echocardiography are usually displayed on separate screens, thus limiting the understanding of the spatial relationships between cardiac devices and cardiovascular structures. In this sense, further advances to refine the management of digital images would allow researchers to improve the creation of fusion images from both fluoroscopy and TEE. This fusion between two imaging modalities in one 3D space and time will create sophisticated images that can be shown on a single screen and better utilized by the cardiologists. Another frequent limitation with 3D images is related to the artifacts that may occur when the ultrasound beam intersects catheters and devices. Additional limitations include the lack of a standardized core curriculum for SHI imagers and the lack of unified guidelines for the use of 3D-TEE during SHI; however, efforts are being made to establish a current state-of-the-art regarding the use of imaging techniques during SHI, including the use of new imaging technologies [59]. Lastly, concerns regarding TEE-related complications during SHI guidance have been recently reported [7,8]. These complications could be associated with the constant manipulation of the probe during SHI. The use of novel techniques, including 3D reconstructions, could reduce the need for excessive probe manipulations during these interventions. To evaluate if these new practices could minimize the risk for TEE-related complications could be a future field of study. 

## 5. Conclusions

The use of 3D-TEE imaging during SHI has several benefits, including an easier localization of the anatomical structures of interest and devices, an easier navigation inside the heart chambers, and a more precise localization of the landing zone of devices. As the use of this technology is expected to extensively increase during transcatheter interventions, appropriate and consistent recommendations for intraprocedural guidance are critical. Future large-scale prospective studies are still needed to demonstrate that the use of 3D-TEE in this scenario generates practical advantages such as shortening of the procedural time, increased procedural success, reduction in procedural complications, and reduced radiation and contrast exposure.

## Data Availability

No new data were created or analyzed in this study. Data sharing is not applicable to this article.
